# Supplementary material for: SUCLA2 mutations cause global protein succinylation contributing to the pathomechanism of a hereditary mitochondrial disease
Source: Nat Commun. 2020 Nov 23;11:5927. doi: 10.1038/s41467-020-19743-4 (PMC7684291; doi:10.1038/s41467-020-19743-4)
Supplement: Supplementary file 1 — Supplementary Information [file 41467_2020_19743_MOESM1_ESM.pdf]

SUPPLEMENTARY INFORMATION

SUCLA2 mutations cause global protein succinylation contributing to the pathomechanism of a hereditary mitochondrial disease

Philipp Gut, Sanna Matilainen, Jesse G. Meyer et al.

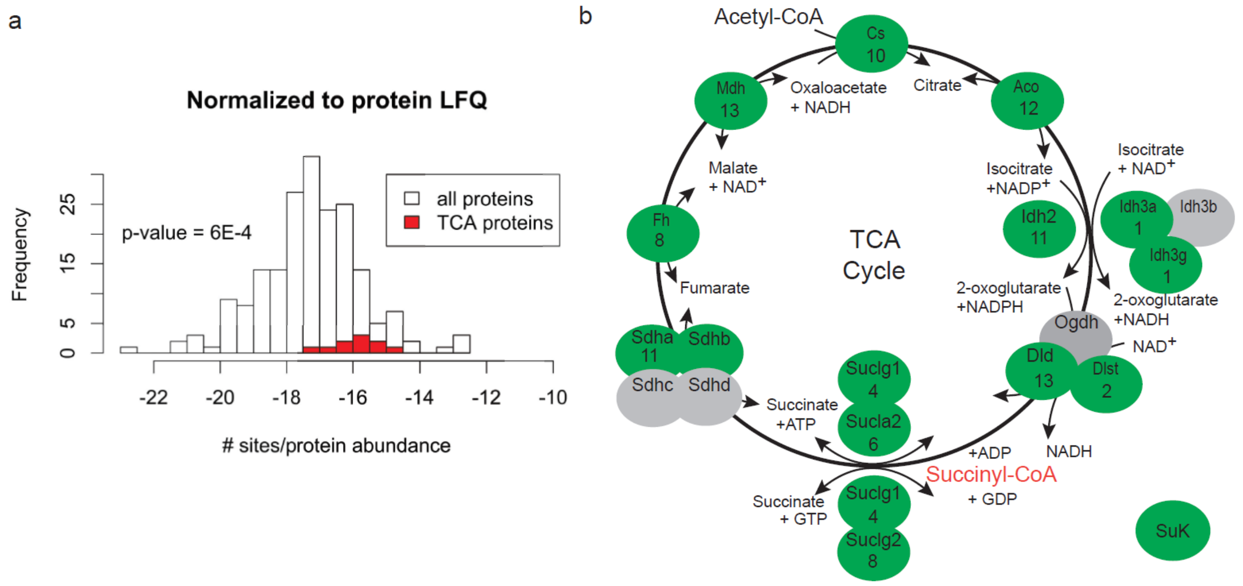

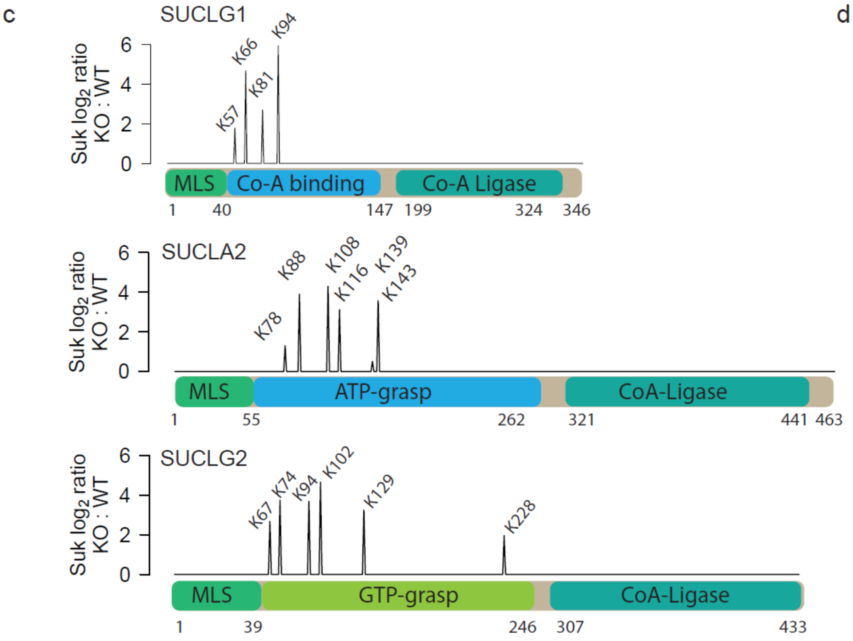

d

Lysine	Fold-Change	P-value
**SUCLG1**		
K57	3.5	1.8E-04
K66	25.6	1.9E-07
K81	6.5	1.8E-08
K94	61.6	4.1E-06
**SUCLA2**		
K78	2.5	5.8E-05
K88	15	2.1E-07
K108	20.4	2.3E-08
K116	8.8	3.0E-09
K139	1.4	3.6E-03
K143	11.9	1.0E-06
**SUCLG2**		
K67	6.4	1.3E-08
K74	13.5	9.3E-07
K94	12.9	2.8E-07
K102	25.3	2.8E-06
K129	9.5	1.4E-06
K228	3.9	1.3E-03

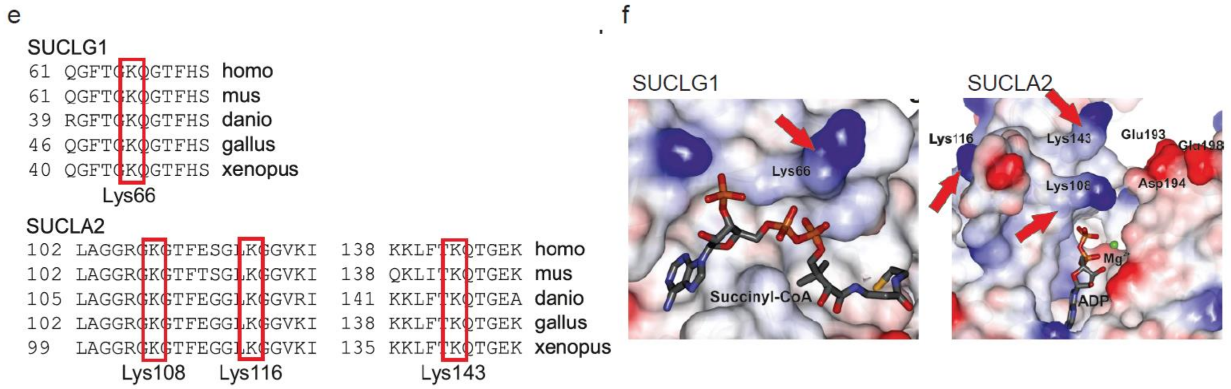

Supplementary Fig. 1 Lysine succinylation sites of the succinyl-CoA ligase complex

a Frequency distribution of number of SuK sites divided by the measured protein abundances. TCA cycle proteins show a significant enrichment towards a higher frequency of SuK sites per protein, normalized by relative label-free quantification (LFQ) to account for bias in protein abundance. Significance in group difference calculated by Wilcoxon sum rank test (p-value = 6E-4) **b** Scheme of the TCA cycle highlighting the number of succinylation sites of each TCA cycle protein in green. **c** Position and fold-change of each succinylation site on the three members of the SCL complex. **d** Fold-change and p-value of each succinylated site affecting the SCL complex. **e** Hyper-succinylated lysine residues are highly conserved among species (Lys66 in SUCLG1 and Lys108, Lys116 and Lys143 in SUCLA2). Statistical significance was calculated using a two-tailed, unpaired student t-test **f** Predicted molecular surface charge distribution of proteins SUCLG1 and SUCLA2 showing the locations of hypersuccinylated lysine residues relative to bound substrate molecules. Succinylation of Lys66 in SUCLG1 and Lys108, 116, and 143 in SUCLA1 indicated by arrows (negatively charged moieties appear in red and positively charged in blue). Source data are provided as a Source Data file.

a

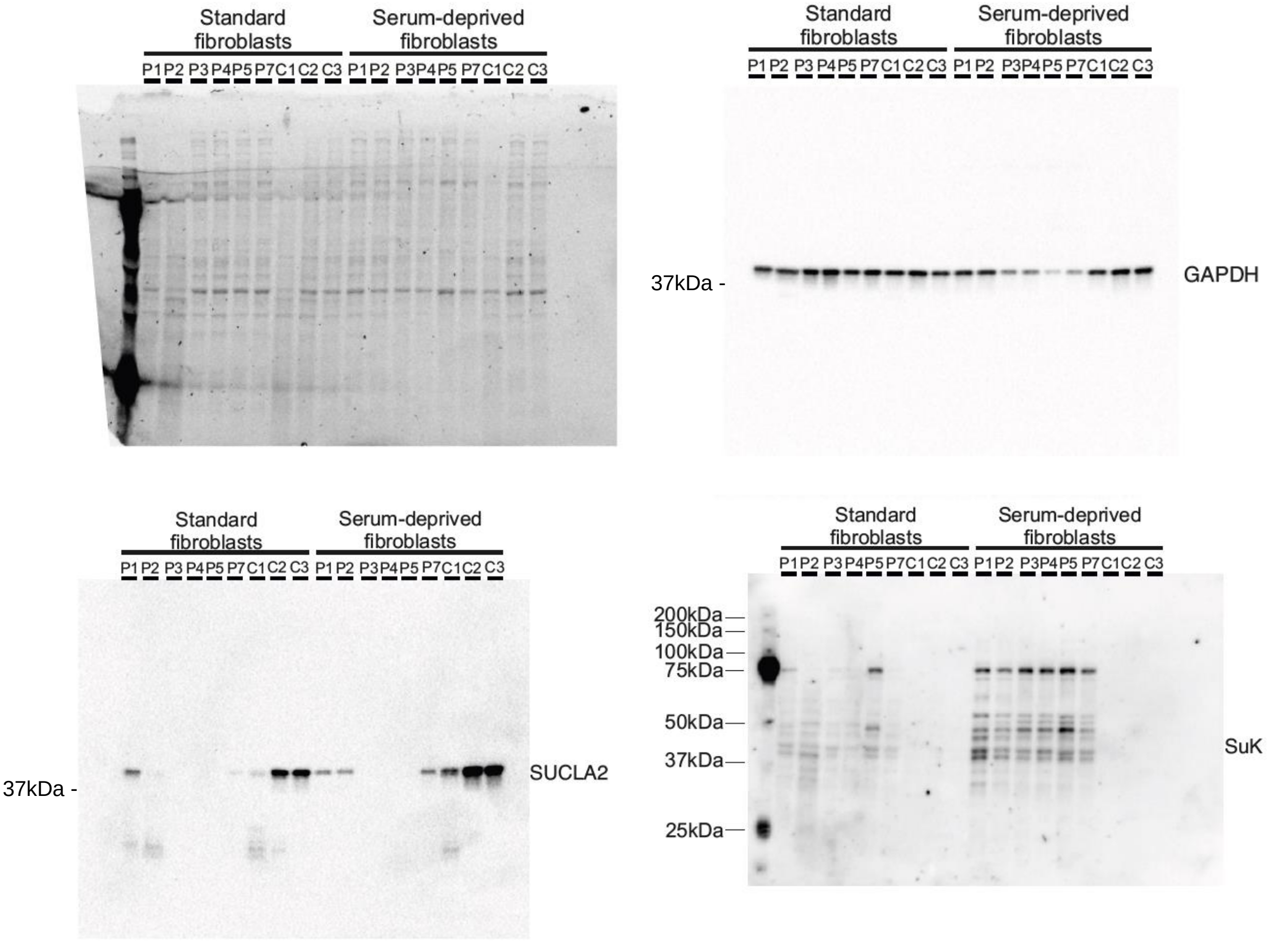
Standard fibroblasts
Serum-deprived fibroblasts
P1 P2 P3 P4 P5 P7 C1 C2 C3 P1 P2 P3 P4 P5 P7 C1 C2 C3
37kDa -
GAPDH
SUCLA2
200kDa
150kDa
100kDa
75kDa
50kDa
37kDa
25kDa
SuK

b

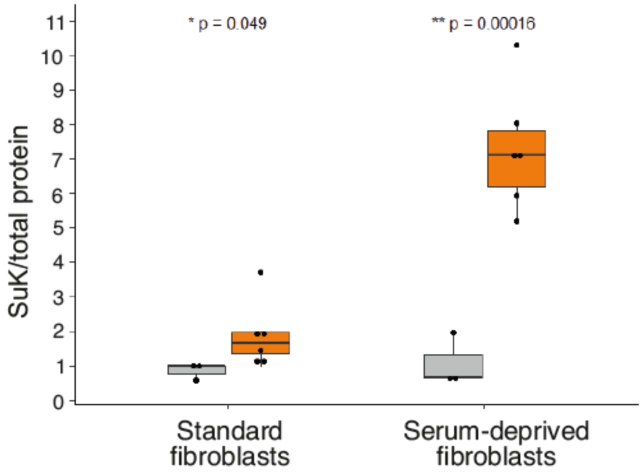
* p = 0.049
** p = 0.00016
SuK/total protein
0
1
2
3
4
5
6
7
8
9
10
11
Standard fibroblasts
Serum-deprived fibroblasts

Supplementary Fig. 2 Lysine succinylation in SCL patient fibroblasts: full blots and quantification

Six SCL patient fibroblast lines and three fibroblast lines from control patients across two different culture conditions (i.e., standard fibroblast culture and serum deprived) were analyzed: full blots and quantification from blots in Figure 1d. Fibroblasts are cultured in standard culture conditions (proliferative condition; indicated as standard fibroblasts) and cells cultured for 5 days in low-serum conditions (non-proliferative condition; indicated as serum-deprived fibroblasts). Patients (P1–P7) carry disease-causing mutations in *SUCLA2*. Controls (C1–C3) are fibroblasts from age-matched patients with other mitochondrial diseases.

a Western blot analysis of fibroblasts showing full blots and molecular mass markers: stain-free SDS-gel for total protein detection (top left panel), a control protein GAPDH (top right), SUCLA2 (bottom left) and anti-succinyllysine antibody, showing molecular mass markers (bottom right).

b Relative succinylation in patient fibroblasts under two different culture conditions: succinylation signal (whole lane) in a and Figure 1E quantified against total protein amount in stain-free gel, shown as a boxplot. On average, in fibroblasts under standard culture, succinylation increased 1.9-fold with $p = 0.048$ in patients vs control patients, and 7.3-fold with $p = 0.00016$ in serum-deprived fibroblasts. There was no significant change in succinylation between control fibroblasts in the two culture conditions. The boxplot shows the median, the first to third quartile, the 1.5x interquartile ranges, and outliers. The p-values were calculated with Welch two-sample t-test assuming unequal variance. Gray: controls, orange: SCL-deficiency patients. Samples sizes: controls, n=3 independent cell lines; SCL-deficiency patients, n=6 independent cell lines. This supplementary figure supports the findings displayed in Figure 1.

Antibodies used are described in detail in Table 1 of the supplementary information as well as in the source data file in the sheets corresponding to Figure 1.

a

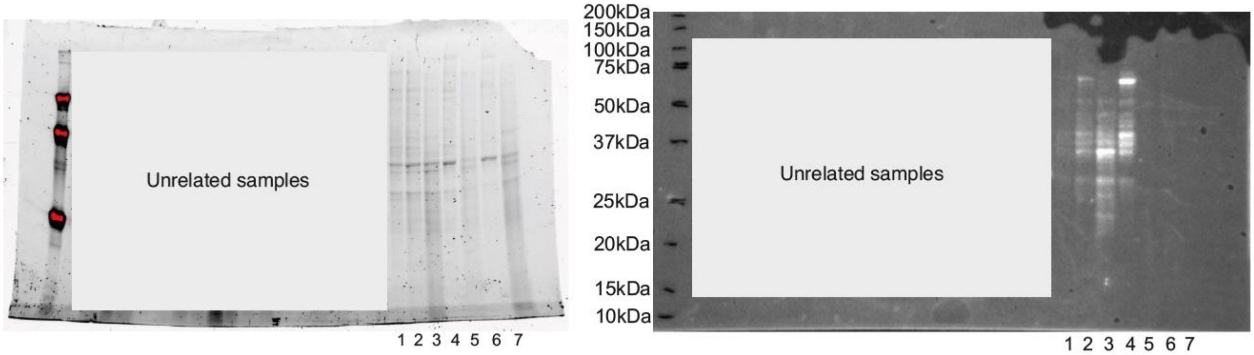

Unrelated samples
1 2 3 4 5 6 7
200kDa
150kDa
100kDa
75kDa
50kDa
37kDa
25kDa
20kDa
15kDa
10kDa
Unrelated samples
1 2 3 4 5 6 7

b

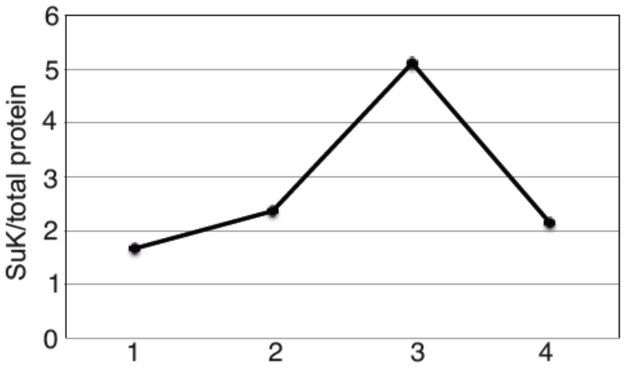

SuK/total protein
0
1
2
3
4
5
6
1
2
3
4

Supplementary Fig. 3 Lysine succinylation in control and SCL patient myoblast cell lines: full blots and quantification

Analysis of lysine succinylation during myoblast differentiation into myotubes of patient with SCL deficiency due to Asp333Gly mutation in *SUCLA2* (P1): Full blots and quantification from Figure 1e. Control: age-matched patient with a non-mitochondrial disease. Samples 1–4: sample 1 is patient myoblasts cultured in standard conditions, sample 2 after 2 days in differentiation culture, sample 3 after 4 days in differentiation culture, and sample 4 fully differentiated myotubes (6 days in differentiation culture); Samples 5–7: cells of control patient during differentiation of myoblasts into myotubes, sample 5 was myoblasts cultured in standard conditions, sample 6 after 2 days in differentiation culture, and sample 7 fully differentiated myotubes (4 days in differentiation culture).

a Left panel: total protein (stain-free SDS-gel) of cells collected at different stages of differentiation of myoblasts into myotubes. Right panel: western blot using anti-succinyllysine antibody showing molecular mass markers in kDa.

b Quantification of protein levels from western blot of panel a of this figure and Figure 1e. Succinylation signal (total signal in lane) compared to total protein amount in stain-free gel shown as line graph. Patient samples (samples 1–4 from 1 SCL deficient patient) are normalized against control myoblasts (from 1 control subject).

This supplementary figure supports the findings displayed in figure 1. Antibodies used are described in detail in Table 1 of the supplementary information as well as in the source data file in the sheets corresponding to Figure 1.

a

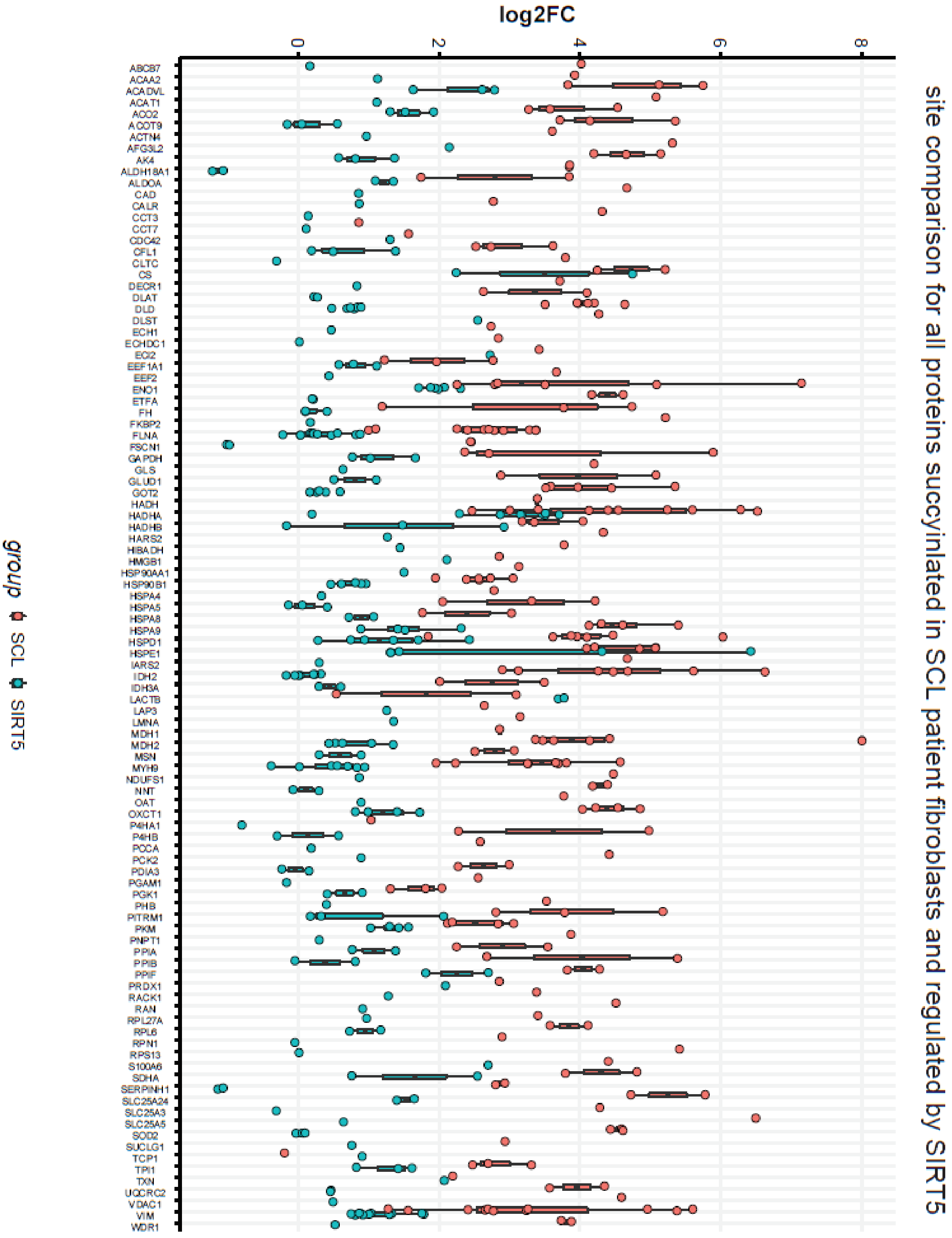
site comparison for all proteins succyinlated in SCL patient fibroblasts and regulated by SIRT5
log2FC
0
2
4
6
8
group
SCL
SIRT5
ABCB7
ACAA2
ACADVL
ACAT1
ACO2
ACOT9
ACTN4
AFG3L2
AK4
ALDH18A1
ALDOA
CAD
CALR
CCT3
CCT7
CDC42
CFL1
CLTC
CS
DECR1
DLAT
DLD
DLST
ECH1
ECHDC1
ECI2
EEF1A1
EEF2
ENO1
ETFA
FH
FKBP2
FLNA
FSCN1
GAPDH
GLS
GLUD1
GOT2
HADH
HADHA
HADHB
HARS2
HIBADH
HMGB1
HSP90AA1
HSP90B1
HSPA4
HSPA5
HSPA8
HSPA9
HSPD1
HSPE1
IARS2
IDH2
IDH3A
LACTB
LAP3
LMNA
MDH1
MDH2
MSN
MYH9
NDUFS1
NNT
OAT
OXCT1
P4HA1
P4HB
PCCA
PCK2
PDIA3
PGAM1
PGK1
PHB
PITRM1
PKM
PNPT1
PPIA
PPIB
PPIF
PRDX1
RACK1
RAN
RPL27A
RPL6
RPN1
RPS13
S100A6
SDHA
SERPINH1
SLC25A24
SLC25A3
SLC25A5
SOD2
SUCLG1
TCP1
TPI1
TXN
UQCRC2
VDAC1
VIM
WDR1

B

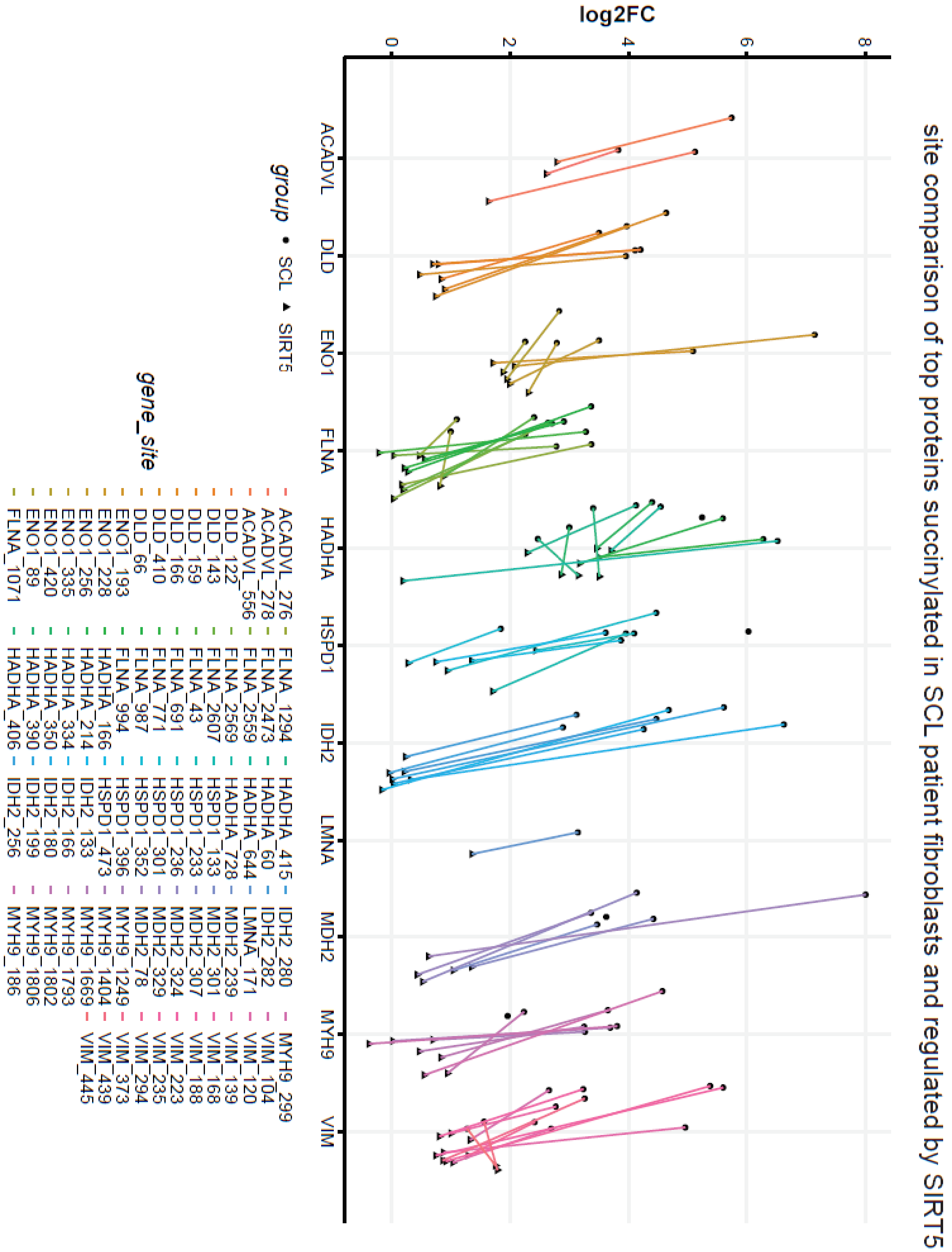
site comparison of top proteins succinylated in SCL patient fibroblasts and regulated by SIRT5
log2FC
0
2
4
6
8
ACADVL
DLD
ENO1
FLNA
HADHA
HSPD1
IDH2
LMNA
MDH2
MYH9
VIM
group • SCL ▲ SIRT5
gene_site
ACADVL_276
ACADVL_278
ACADVL_556
DLD_122
DLD_143
DLD_159
DLD_166
DLD_410
DLD_66
ENO1_193
ENO1_228
ENO1_256
ENO1_335
ENO1_420
ENO1_89
FLNA_1071
FLNA_1294
FLNA_2473
FLNA_2559
FLNA_2569
FLNA_2607
FLNA_43
FLNA_691
FLNA_771
FLNA_987
FLNA_994
HADHA_166
HADHA_214
HADHA_334
HADHA_350
HADHA_390
HADHA_406
HADHA_415
HADHA_60
HADHA_644
HADHA_728
HSPD1_133
HSPD1_233
HSPD1_236
HSPD1_301
HSPD1_352
HSPD1_396
HSPD1_473
IDH2_133
IDH2_166
IDH2_180
IDH2_199
IDH2_256
IDH2_280
IDH2_282
LMNA_171
MDH2_239
MDH2_301
MDH2_307
MDH2_324
MDH2_329
MDH2_78
MYH9_1249
MYH9_1404
MYH9_1669
MYH9_1793
MYH9_1802
MYH9_1806
MYH9_186
MYH9_299
VIM_104
VIM_120
VIM_139
VIM_168
VIM_188
VIM_223
VIM_235
VIM_294
VIM_373
VIM_439
VIM_445

Supplementary Fig. 4 Comparison of individual sites between SIRT5 and SCL deficiencies

a Comparison of log2(fold change) of succinyl-lysine sites from proteins detected in both SUCLA2 deficient patient fibroblasts and *Sirt5*$^{-/-}$ fibroblasts relative to controls. **b** Site comparison of top 11 proteins from the SCL datasets with lines connecting each site. Because the data from SUCLA2 deficiency–dependent succinylation were from human samples and *Sirt5*$^{-/-}$ data were from a prior publication using mouse samples, sequence alignment was carried out to ensure all possible succinylation sites were matched between datasets. Source data are provided as a Source Data file.

***sucla2* loss of function**

Exon 3 11-bp deletion

5'wt<br>5'mut	GTTCTAAAGACTTGGTTATAAAAGCCCAAGTGCTGGCGGGTGGTCGAGGCAAAGGCACTTTTGAAGGA<br>GTTCTAAAGACTTGGTTATAAAAGCCCAAGTGCTGGCGGGTGGTCGAGGCAAAGGCACTTTTGA----
5'wt<br>5'mut	GGACTGAAAGGAGGAGTCAGAATCGTTTACTC<br>-------AAGGAGGAGTCAGAATCGTTTACTC

***sirt5* loss of function**

Exon 5 13-bp deletion

5'wt<br>5'mut	GTAATGCGCAGTAAGATGCCGAATCCAGCACATCTGGCTATAGCAGAGTGTGAGGCTCGTCTCGGCCA<br>GTAATGCGCAGTAAGATGCCGAATCCAGCACATCTGGCTATAGCAGAGTGTGAGGCTCGTCTCGGCCA
5'wt<br>5'mut	GCAGGGGCGCTCTGTGGTGATCATCACCCAAAACATTGATGAACTGCACCATCGGGCTGGGTCTAAAC<br>GCAGGGGCGCTCTGTGGTGATCATCACCCAAAACATTGATGAACTG-------------GGTCTAAAC
5'wt<br>5'mut	ATGTCTATGAGATCCAC<br>ATGTCTATGAGATCCAC

Supplementary Fig. 5 cDNA alignments of wild-type and mutant sequences

Alignments of wild-type and mutant sequences of the exons targeted with Crispr/Cas9 to generate loss-of-function zebrafish mutants. Source data are provided as a Source Data file.

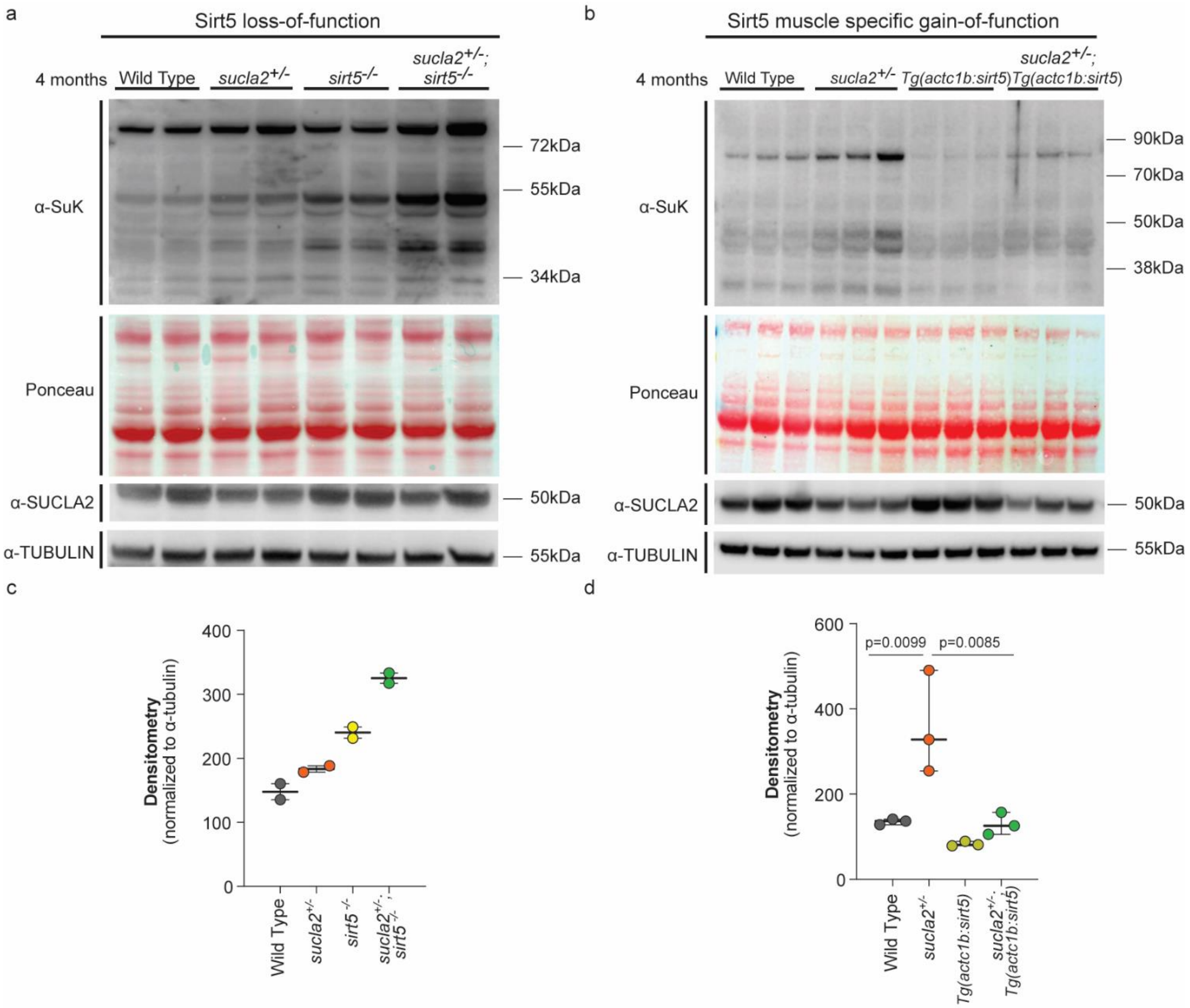

a
Sirt5 loss-of-function
4 months
Wild Type
sucla2+/-
sirt5-/-
sucla2+/-; sirt5-/-
α-SuK
72kDa
55kDa
34kDa
Ponceau
α-SUCLA2
50kDa
α-TUBULIN
55kDa
b
Sirt5 muscle specific gain-of-function
4 months
Wild Type
sucla2+/-
Tg(actc1b:sirt5)
sucla2+/-; Tg(actc1b:sirt5)
α-SuK
90kDa
70kDa
50kDa
38kDa
Ponceau
α-SUCLA2
50kDa
α-TUBULIN
55kDa
c
Densitometry (normalized to α-tubulin)
400
300
200
100
0
Wild Type
sucla2+/-
sirt5-/-
sucla2+/-; sirt5-/-
d
Densitometry (normalized to α-tubulin)
600
400
200
0
p=0.0099
p=0.0085
Wild Type
sucla2+/-
Tg(actc1b:sirt5)
sucla2+/-; Tg(actc1b:sirt5)

Supplementary Fig. 6 Sirt5 modulates protein succinylation in skeletal muscle of *sucla2*$^{+/-}$ zebrafish

a Western blot analysis of global protein succinylation in muscle tissue extracted from 4-month-old zebrafish with a heterozygous mutation in *sucla2* and a homozygous mutation in *sirt5*, combined gene deficiencies, and controls**.** (n=2 per genotype, adult skeletal muscle from individual zebrafish). **b** Pan-succinyl-lysine western blots of muscle samples extracted from 4-month-old adults overexpressing *Tg(actc1b:*sirt5*;cryaa*zsgreen) in wild-type or *sucla2*$^{+/-}$ animals (n=2 per genotype, adult skeletal muscle from individual zebrafish).

Pan-succinyl-lysine antibodies were used to mark lysine succinylation, and tubulin antibodies were used as loading controls. Ponceau staining serves as an additional loading control. Images from western blot experiments are derived from a single experiment.

c and **d** Quantification of the pan-succinyl-lysine western blots by Image J using the mean of the densitometry of the three main bands, normalized to tubulin. The boxplots show the median, the first to third quartile, and minima and maxima) **d** Ordinary one-way Anova with multiple comparisons tests (Tukey) was used for statistical analysis. Source data are provided as a Source Data file.

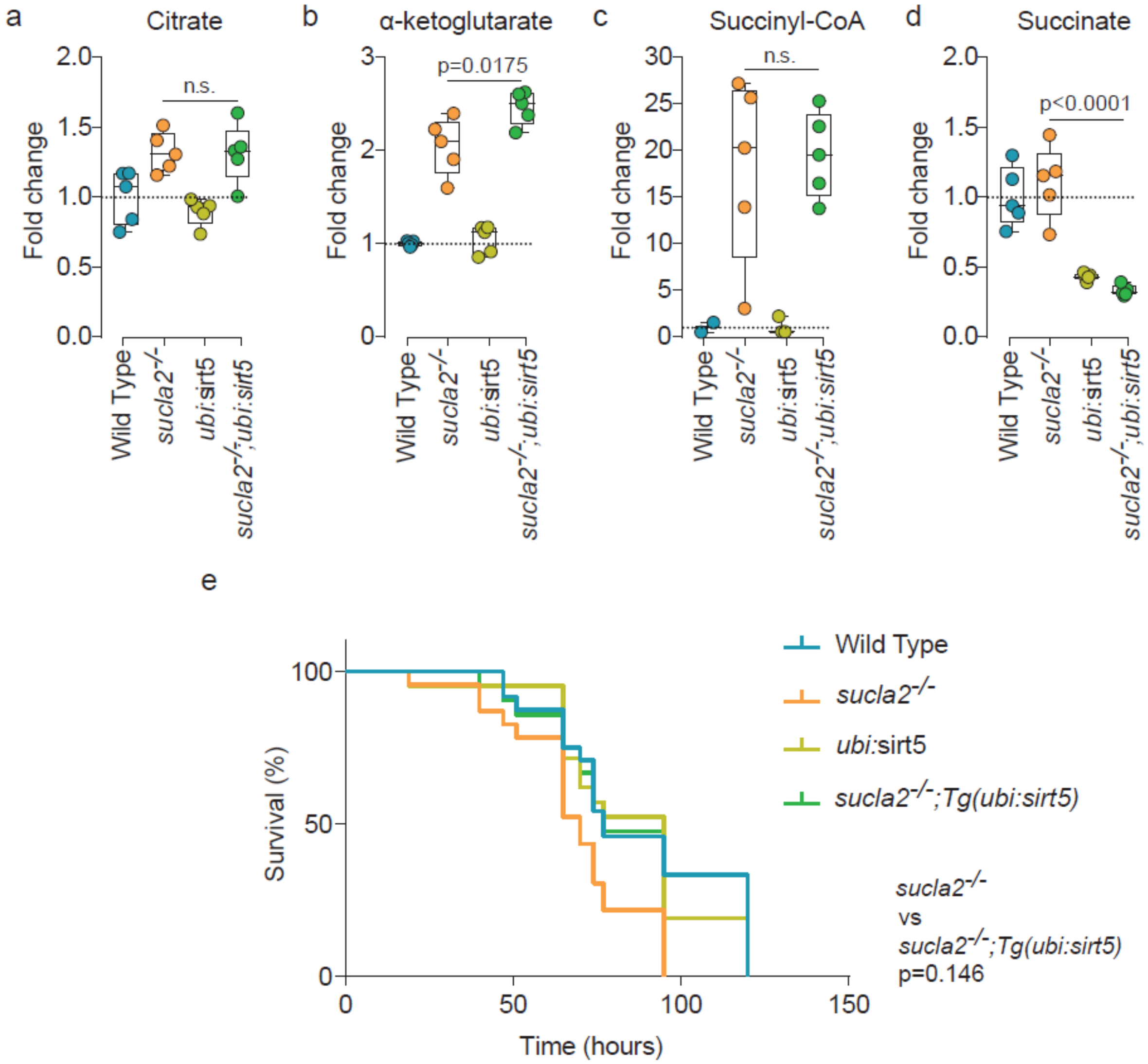
a
Citrate
Fold change
n.s.
Wild Type
sucla2-/-
ubi:sirt5
sucla2-/-;ubi:sirt5
b
α-ketoglutarate
p=0.0175
c
Succinyl-CoA
n.s.
d
Succinate
p<0.0001
e
Survival (%)
Time (hours)
Wild Type
sucla2-/-
ubi:sirt5
sucla2-/-;Tg(ubi:sirt5)
sucla2-/-
vs
sucla2-/-;Tg(ubi:sirt5)
p=0.146

Supplementary Fig. 7 Effects of *sirt5* overexpression on energy metabolism in *sucla2*$^{-/-}$ zebrafish

a-d Relative levels of TCA cycle metabolites in wild-type and *sucla2*$^{-/-}$ animals with and without overexpression of *sirt5*. Eight larvae were pooled at 7 dpf and processed for metabolite analysis (n=5 pools of 8 larvae each). Ordinary one-way Anova with multiple comparisons tests (Tukey) was used for statistical analysis. n.s., not significant. **e** Survival of *sucla2-/-* and control zebrafish larvae with or without *sirt5* overexpression in volume-restricted conditions (*sucla2*$^{+/+}$, n=24; *sucla2*$^{-/-}$, n=23; *sucla2*$^{+/+}$*;ubi:*sirt5, n=21; *sucla2*$^{-/-}$;*ubi:*sirt5, n=21. N indicate the number of single zebrafish larvae used for each genotype). The boxplots show the median, the first to third quartile, and minima and maxima). Pairwise comparison using conventional rog-Rank test. P-value adjustment was performed by Bonferroni correction. Source data are provided as a Source Data file.

a

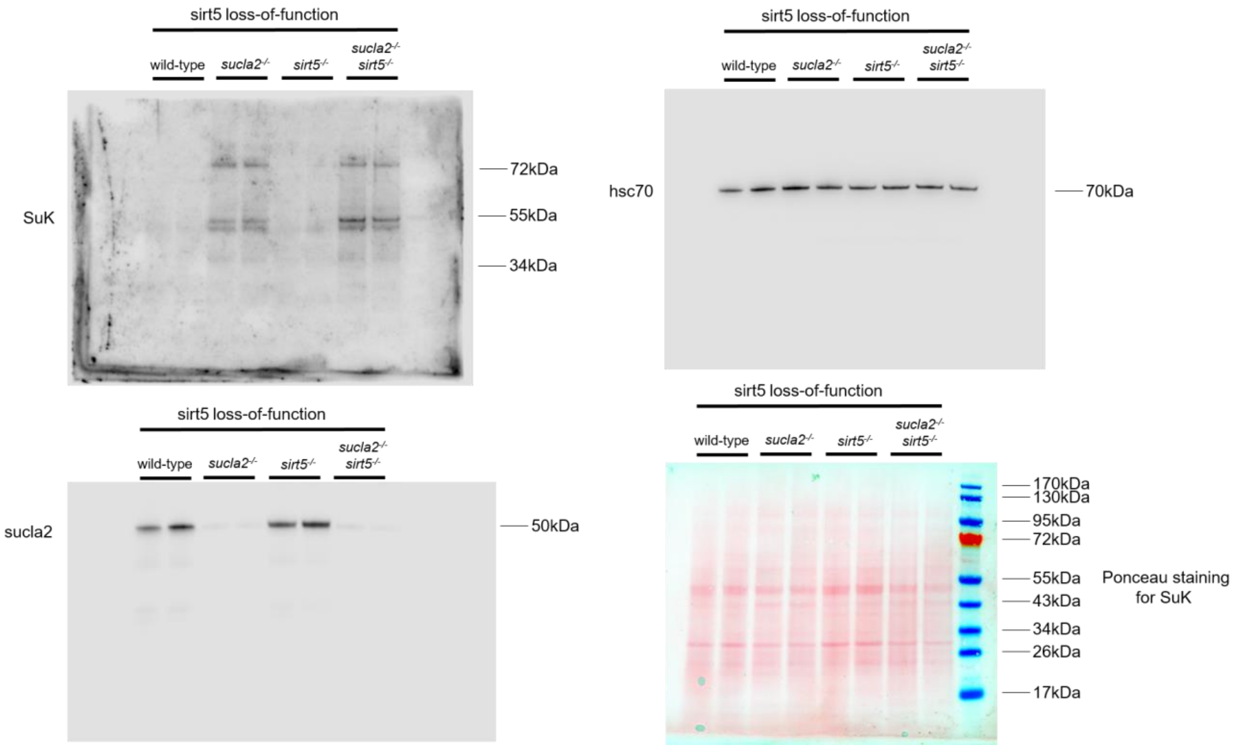
sirt5 loss-of-function
wild-type
sucla2-/-
sirt5-/-
sucla2-/- sirt5-/-
SuK
72kDa
55kDa
34kDa
hsc70
70kDa
sucla2
50kDa
170kDa
130kDa
95kDa
72kDa
55kDa
43kDa
34kDa
26kDa
17kDa
Ponceau staining for SuK

b

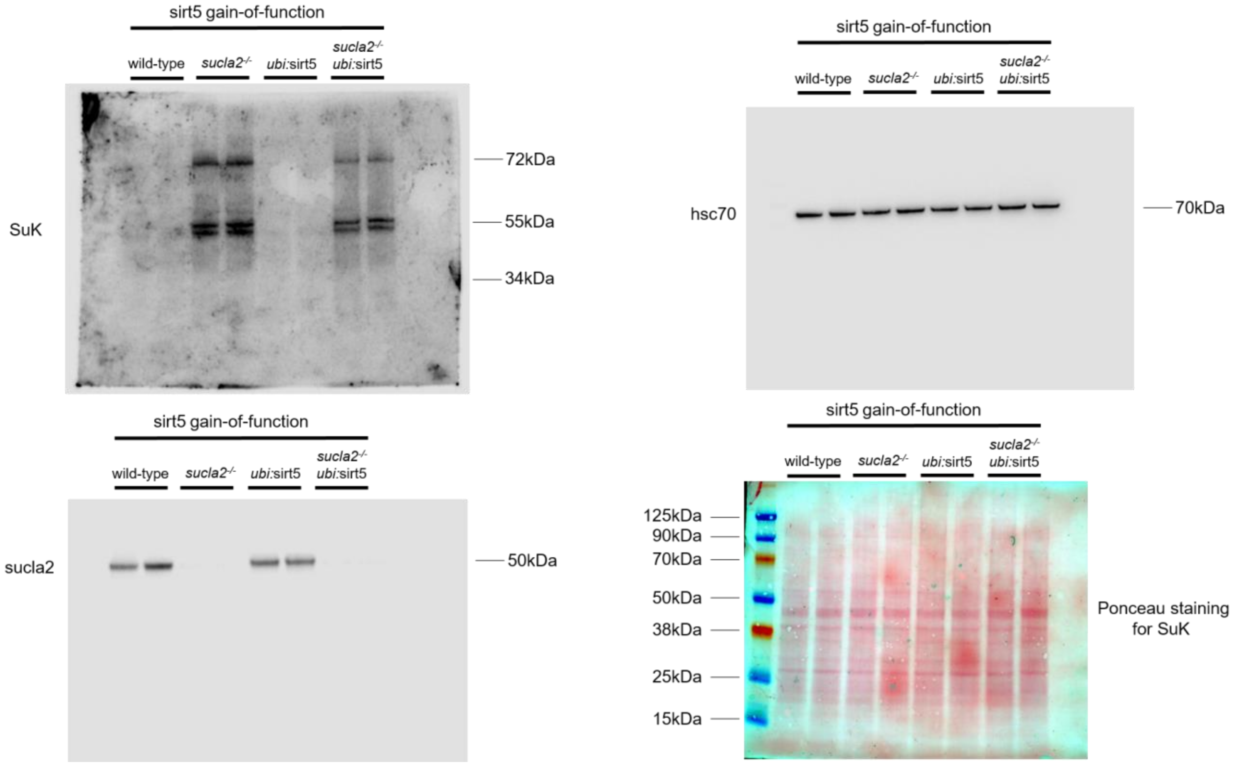
sirt5 gain-of-function
wild-type
sucla2-/-
ubi:sirt5
sucla2-/- ubi:sirt5
SuK
72kDa
55kDa
34kDa
hsc70
70kDa
sucla2
50kDa
125kDa
90kDa
70kDa
50kDa
38kDa
25kDa
15kDa
Ponceau staining for SuK

c

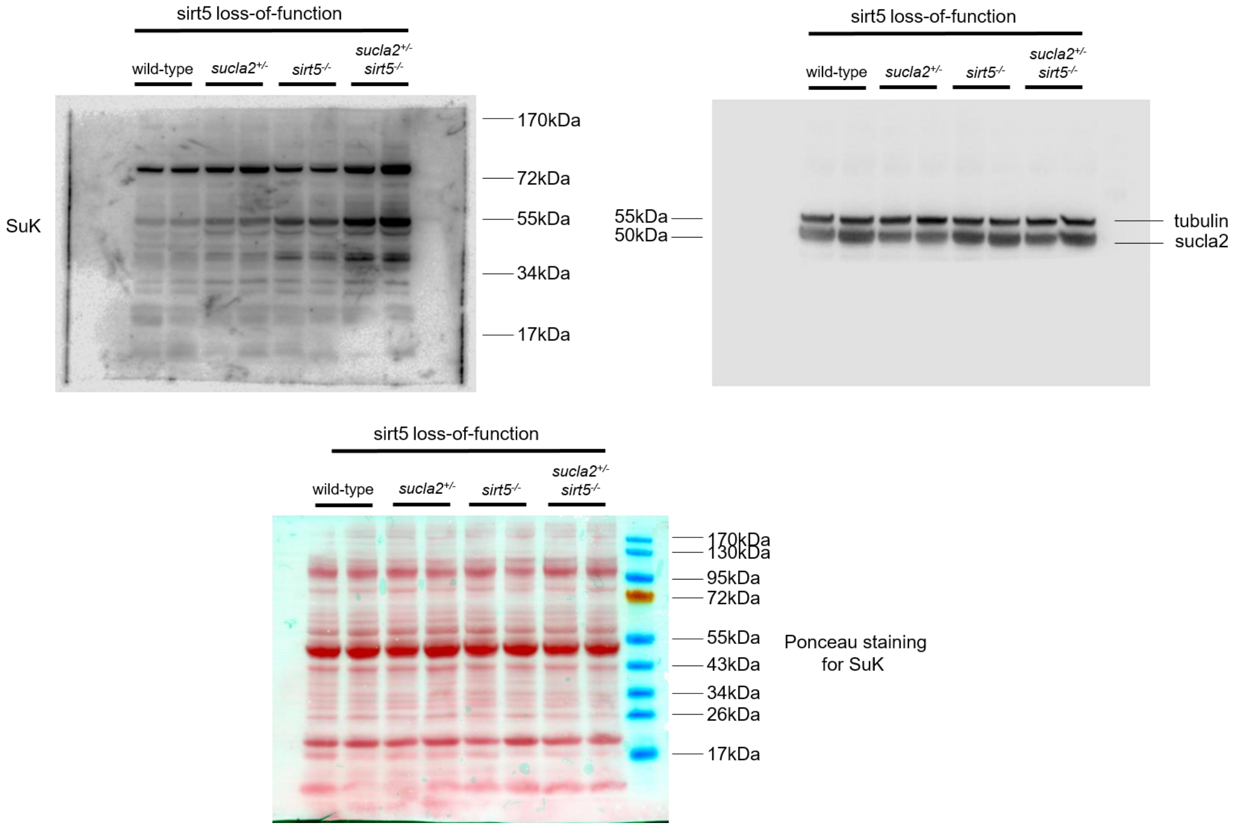
sirt5 loss-of-function
wild-type
sucla2+/-
sirt5-/-
sucla2+/- sirt5-/-
SuK
170kDa
72kDa
55kDa
34kDa
17kDa
sirt5 loss-of-function
wild-type
sucla2+/-
sirt5-/-
sucla2+/- sirt5-/-
55kDa
50kDa
tubulin
sucla2
sirt5 loss-of-function
wild-type
sucla2+/-
sirt5-/-
sucla2+/- sirt5-/-
170kDa
130kDa
95kDa
72kDa
55kDa
43kDa
34kDa
26kDa
17kDa
Ponceau staining for SuK

d

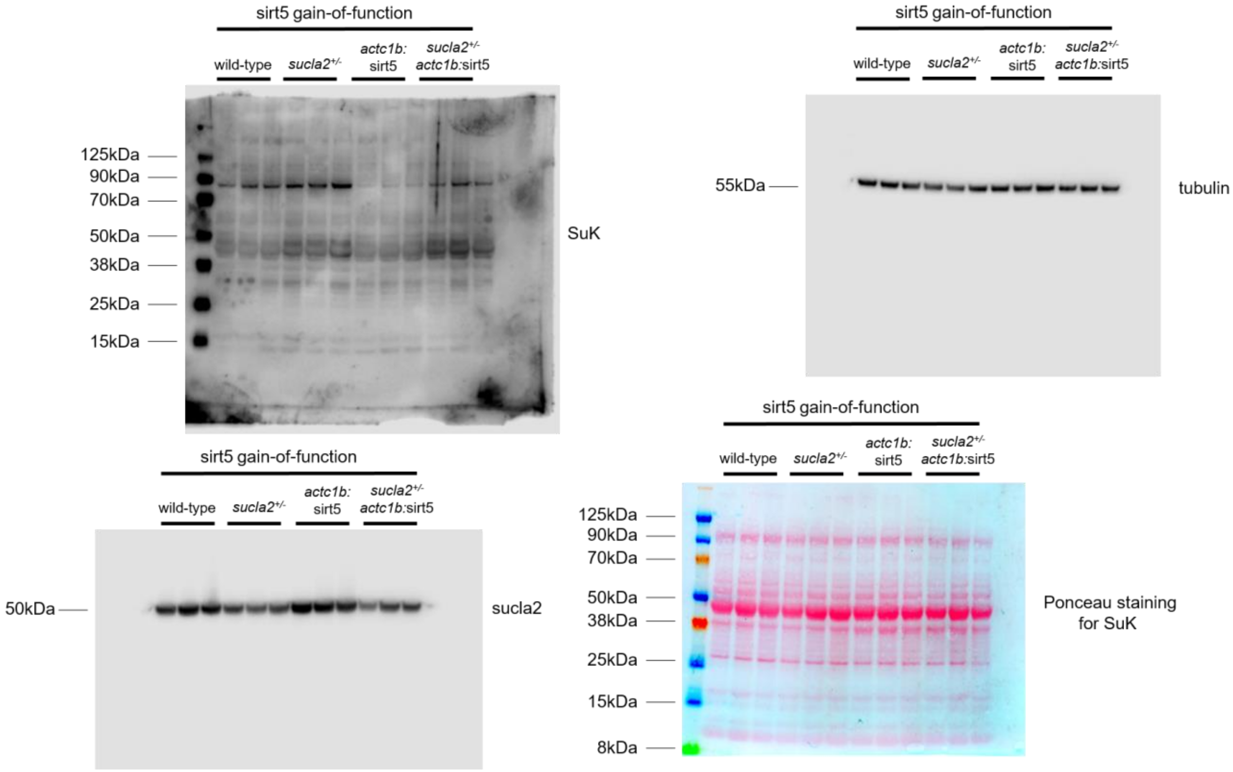

Supplementary Fig. 8 Lysine succinylation in zebrafish: full blots

a Western blot analysis of protein extracts from zebrafish larvae with homozygous mutations in *sucla2*, *sirt5*, or combined gene deficiencies and controls (7 dpf, pools of 10–15 whole larvae), showing full blots: global protein succinylation (top left panel), Hsc70 as loading control (top-right), α-Sucla2 (bottom left) and ponceau staining for total protein detection, showing molecular mass markers (bottom right). **b** Western blot analysis of protein extracts from zebrafish larvae overexpressing *sirt5* in wild-type or *sucla2*$^{-/-}$ animals (7 dpf, pools of 10–15 larvae), showing full blots: global protein succinylation (top left panel), **c** Western blot analysis a control protein Hsc70 (top right), α-Sucla2 (bottom left) and ponceau staining for total protein detection, showing molecular mass markers (bottom right) of protein extracts from 4-month-old zebrafish muscle with heterozygous mutation in *sucla2* and homozygous in *sirt5*, or combined gene

deficiencies and controls (n=2), showing full blots: global protein succinylation (top left panel), a control protein α-tubulin and α-sucla2 (top right) and ponceau staining for total protein detection, showing molecular mass markers (bottom). **d** Western blot analysis of protein extracts from 4-month-old zebrafish muscle, overexpressing *sirt5* specifically in muscle in wild-type or *sucla2*$^{-/-}$ animals (n=3), showing full blots: global protein succinylation with molecular mass markers (top left panel), a control protein α-tubulin (top right), α-Sucla2 (bottom left) and ponceau staining for total protein detection, showing molecular mass markers (bottom right). This supplementary figure supports the findings displayed in figure 4. Antibodies used are described in detail in Table 1 of the supplementary information as well as in the source data file in the sheets corresponding to Figure 4.

Supplementary Methods

Mass Spectrometric Analysis

All mass spectrometric data was collected using either a SCIEX TripleTOF 6600 or a TripleTOF 5600 system, each coupled to an Eksigent nanoflow liquid chromatography pump and a cHiPLC chromatography system. For different sample types, such as fibroblasts and myotubes, respectively, and for digested cell lysates and enriched succinylated peptides, respectively, different (optimized) experimental parameters were used for the HPLC-MS system as outlined below.

Chromatography

Samples were analyzed by reverse-phase HPLC-ESI-MS/MS using an Eksigent Ultra Plus nano-LC 2D HPLC system (Dublin, CA) with a cHiPLC system (Eksigent) which was directly connected to a quadrupole time-of-flight (QqTOF) TripleTOF 6600 (or TripleTOF 5600) mass spectrometer (SCIEX, Concord, CAN). After injection, peptide mixtures were loaded onto a C18 pre-column chip (200 µm x 0.4 mm ChromXP C18-CL chip, 3 µm, 120 Å, SCIEX) and washed at 1-2 µl/min for 10 min with the loading solvent (H_2O/0.1% formic acid) for desalting. Subsequently, peptides were transferred to the 75 µm x 15 cm ChromXP C18-CL chip, 3 µm, 120 Å, (SCIEX), and eluted at a flow rate of 300 nL/min with the following gradients using aqueous (A) and acetonitrile (B) solvent buffers.

i) *myotubes – whole cell lysate* (240 min gradient): linear gradient from 5% to 28% mobile phase B over 200 min; mobile phase B was then ramped to 80% over 5 minutes, held at 80% B for 8 minutes, before returning to 5% B for 26 min re-equilibration.

ii) *fibroblasts – whole cell lysate* (240 min gradient): linear gradient from 5% to 30% mobile phase B over 200 min; mobile phase B was then ramped to 90% over 1 minutes, held at 90% B for 10 minutes before returning to 5% B for 28 min re-equilibration.

iii) *myotubes – PTM enriched peptides* (180 min gradient): keep at 5% B for 5 min; linear gradient from 5% to 8% mobile phase B over 15 min; linear gradient from 8% to 35% mobile phase B over 97 min; mobile phase B was then ramped to 80% over 20 minutes, held at 80% B for 10 minutes, before returning to 5% B for 30 min re-equilibration.

iv) *fibroblasts – PTM enriched peptides* (120 min gradient): linear gradient from 5% to 35% mobile phase B over 80 min; mobile phase B was then ramped to 80% over 5 minutes, held at 80% B for 8 minutes before returning to 5% B for 25 min re-equilibration.

Data-dependent acquisition (DDA) was used to identify peptides. Every cycle consisted of one 250 ms precursor ion scan followed by isolating the top ‘n’ most abundant precursor ions (at 1 m/z resolution) for collision induced dissociation tandem mass spectrometry (MS/MS) in ‘high sensitivity’ product ion scan mode, as detailed more specifically below for the different samples and experiments (software: Analyst 1.7; build 96).

i) *TripleTOF 6600: myotubes – PTM enriched peptides.* To build a spectral library for PTM enriched peptides, the mass spectrometer was operated in DDA mode. Every cycle consisted of one 250 ms precursor ion scan followed by isolating the top 30 most abundant precursor ions between 400-1,500 m/z. Tandem mass spectra were accumulated for 50 ms collecting fragment ion masses between 100-2,000 m/z yielding a total cycle time of 1.8 sec.

ii) *TripleTOF 5600: fibroblasts – PTM enriched peptides.* To build a spectral library for PTM enriched peptides, the mass spectrometer was operated in DDA mode. Every cycle consisted of one 250 ms precursor ion scan followed by isolating the top 10 most abundant precursor ions between 400-1,300 m/z. Tandem mass spectra were accumulated for 100 ms collecting fragment ion masses between 100-2,000 m/z yielding a total cycle time of 1.3 sec.

Data-Independent acquisition (DIA) was used to quantify peptides from both PTM-enriched fractions for PTM-site level quantification, as well as from whole cell lysates for protein level quantification (for PTM-site normalization purposes). Every DIA cycle consisted of 250 ms precursor ion scan followed by 64 variable-width isolation windows to produce fragment ion spectra (software: Analyst 1.7; build 96). The 64 variable windows isolation scheme was the same as recently reported, here referred to as 64vw-Scheme1 [1]. In one case (see below) the 64 variable windows were generated using SWATHTuner, here referred to as 64vw-Scheme2 [2]. The 64 variable window isolation schemes 64vw-Scheme1 and 64vw-Scheme2 are available as part of the Skyline documents uploaded to Panorama (https://panoramaweb.org/Schilling/SuccinylCoALigase/project-begin.view?). DIA tandem mass spectra produce complex MS/MS spectra, which are a composite of all the analytes within each selected Q1 m/z window.

i) *TripleTOF 6600: myotubes – PTM enriched peptides.* To quantify the PTM enriched peptides, the mass spectrometer was operated in DIA mode. Every cycle consisted of one 250 ms precursor ion scan with 400-1,250 m/z mass range. Subsequently, windows of variable width are passed in incremental steps over the full mass range (m/z 400-1,250). The cycle time of 3.2 sec

includes the 250 msec precursor ion scan followed by 45 msec accumulation time for each of the 64 DIA segments (64vw-Scheme1) monitoring fragment ion masses between 100-1,500 m/z.

ii) *TripleTOF 5600: fibroblasts – PTM enriched peptides.* To quantify the PTM enriched peptides, the mass spectrometer was operated in DIA mode. Every cycle consisted of one 250 ms precursor ion scan with 400-1,250 m/z mass range. Subsequently, windows of variable width are passed in incremental steps over the full mass range (m/z 400-1,250). The cycle time of 3.0 sec includes the 250 msec precursor ion scan followed by 42 msec accumulation time for each of the 64 DIA segments (64vw-Scheme2) monitoring fragment ion masses between 350-2,000 m/z.

iii) *myotubes – whole cell lysate.* To quantify the protein level changes, the mass spectrometer was operated in DIA mode. Every cycle consisted of one 250 ms precursor ion scan with 400-1,250 m/z mass range. Subsequently, windows of variable width are passed in incremental steps over the full mass range (m/z 400-1,250). The cycle time of 3.2 sec includes the 250 msec precursor ion scan followed by 45 msec accumulation time for each of the 64 DIA segments (64vw-Scheme1) monitoring fragment ion masses between 100-1,500 m/z.

iv) *fibroblasts – whole cell lysate.* To quantify the protein level changes, the mass spectrometer was operated in DIA mode. Every cycle consisted of one 250 ms precursor ion scan with 400-1,250 m/z mass range. Subsequently, windows of variable width are passed in incremental steps over the full mass range (m/z 400-1,250). The cycle time of 3.2 sec includes the 250 msec precursor ion scan followed by 45 msec accumulation time for each of the 64 DIA segments (64vw-Scheme1) monitoring fragment ion masses between 100-2,000 m/z.

Supplementary References

1. Collins, B.C. *et al.* Multi-laboratory assessment of reproducibility, qualitative and quantitative performance of SWATH-mass spectrometry. *Nat Commun* **8**, 291 (2017).
2. Zhang, Y. *et al.* The Use of Variable Q1 Isolation Windows Improves Selectivity in LC-SWATH-MS Acquisition. *J Proteome Res* **14**, 4359-4371 (2015).

Supplementary Table 1 Antibodies and reagents

REAGENT or RESOURCE	SOURCE	IDENTIFIER
**Antibodies and western blotting reagents**		
Pan anti-succinyllysine antibody<br><br>(working dilution 1/1000)	PTM biolabs	CAT# PTM-401
SUCLA2<br><br>(working dilution 1/1000)	Abcam	CAT# ab97868
TUBULIN<br><br>(working dilution 1/10000)	Abcam	CAT# ab6046
HSC-70<br><br>(working dilution 1/5000)	Santa Cruz	CAT# sc-7298
GAPDH<br><br>(working dilution 1/1000)	Cell Signaling Technology	CAT# 2118
PTMScan succinyl-lysine motif [Succ-K] Kit<br><br>(working dilution 1/1000)	Cell Signaling Technology	CAT# 13764
**Chemicals**		
SYBR green master mix	Themo Fisher	CAT#4385614
SDS precast gel	Biorad, Criterion™ TGX Stain-Free	CAT #567-8045
Agarose beads	Cell Signaling Technologies, PTMScan	CAT# 13764
Zebrafish medium	Instant Ocean® sea salt	CAT# SS15-10
Tris (hydoxymethyl) aminomethane	Sigma	CAT# 252859-500G
Sequanal-grade Urea	Thermo Fisher Scientific	CAT# 29700
Halt protease inhibitor	Thermo Fisher Scientific	CAT# 78430
Trichostatin A (5 mM)	Sigma	CAT# T1952
Nicotinamide	Sigma	CAT# N0636-100G
Dithiothreitol	Sigma	CAT# D9779
Iodoacetamide	Sigma	CAT# I1149
Sequencing-grade trypsin, frozen	Promega	CAT# V5113
LC-MS-grade formic acid	Sigma	CAT# 94318
LC-MS-grade trifluoracetic acid	VWR	CAT# 85183
LC-MS-grade methanol	Fisher Scientific	Cat# A456
LC-MS-grade water	Fisher Scientific	Cat# W6
LC-MS-grade ammonium hydroxide	Fisher Scientific	Cat# A470
Acetic acid, glacial	Fisher Scientific	Cat# A38
**Reagents or resources**		
Oasis HLB 1-cc vac cartridges 30 mg	Waters	CAT# 186003908
Empore Sorbent Disks	3M	CAT# 98060402173

2.0-mL microfuge tubes	Thermo Fisher	CAT# 21-402-905
0.65 microcentrifuge tubes	Thermo Fisher	
Oligonucleotides		
HRM primer for *sucla2*$^{-/-}$ genotyping	Invitrogen	5′-CTTGGTTATAAAAGCCCAAGTGC-3′ (forward) 5′- GAGTAAACGATTCTGACTCCTCC-3′ (reverse)
HRM primer for *sirt5*$^{-/-}$ genotyping	Invitrogen	5′- GGGTGGGTAATTGGGAAGTT-3′ (forward) and 5′- GATGGTCCAGTCCTGGTTTG 3′ (reverse)
**Experimental models: cell Lines**		
Patient-derived fibroblasts	University of Helsinki	n/a
Patient-derived myoblasts	University of Helsinki	n/a
Restrictions apply to the availability of the patient derived cell lines: their research use is restricted to the labs that obtained the informed consent from the patients. Additional information regarding the cell lines can be obtained from the lab of origin.		
**Experimental models: zebrafish**		
sucla2$^{-/-}$	Nestlé Institute of Health Sciences	*sucla2*$^{-/-nei010}$
Sirt5$^{-/-}$	Nestlé Institute of Health Sciences	*sirt5*$^{-/-nei004}$
Tg(ubi:sirt5;cryaa:zsgreen1)	Nestlé Institute of Health Sciences	*Tg(ubi:sirt5;cryaa:zsGreen1)*^{nei005}
Tg(actc1bi:sirt5;cryaa:zsgreen1)	Nestlé Institute of Health Sciences	*Tg(actc1b:sirt5;cryaa:zsGreen1)*^{nei006}
All zebrafish lines can be requested for research purposes from the lab of origin		
**Software**		
R (version 3.2.3)	The R Foundation	https://www.r-project.org/
R Studio	The R Foundation	https://www.rstudio.com/
Prism Graphpad Version 8.4.1 (676)	GraphPad	https://praphpad.com/scientific-software/prism/
Discovery Studio v4.1	Accelrys	
MaxQuant v1.6.17.0		https://maxquant.org/
Spectronaut pulsar version 11.0.15038.12.33511	Spectronaut,	https://www.biognosys.com/shop/spectronaut-x
Skyline 20.1		https://skyline.ms/
PIQED version 1.01		https://github.com/jgmeyerucsd/PIQEDia
R scripts		https://github.com/jessegmeyerlab/SUCLA2-deficiency
Sieve 2.0	ThermoFisher Scientific	https://portal.thermo-brims.com/index.php/component/thermosoftwares/thermosoftware/67?Itemid=121
**Deposited data**		
Proteomics raw dataset from patient myotubes and fibroblasts	UCSD proteomics resource massive.	Publicly available data can be found using the following URL: [https://massive.ucsd.edu/ProteoSAFe/dataset.jsp?task=7ca3028f854e4996b58f1fa1fc2286fd]
Peptide spectral library and quantitative chromatograms	Panoramaweb repository software for targeted mass spectrometry assays	Publicly available data can be found using the following URL: [https://panoramaweb.org/project/Schilling/SuccinylCoALigase/begin.view]
Metabolomics data from SCL patient fibroblasts	Metabolomics workbench.	DOI ID: [http://dx.doi.org/10.21228/M8M116] mwTab Identifier: ID ST001441
