# Supplementary material for: SUCLA2 mutations cause global protein succinylation contributing to the pathomechanism of a hereditary mitochondrial disease
Source: Nat Commun. 2020 Nov 23;11:5927. doi: 10.1038/s41467-020-19743-4 (PMC7684291; doi:10.1038/s41467-020-19743-4)
Supplement: Supplementary file 3 — Description of Additional Supplementary Files [file 41467_2020_19743_MOESM3_ESM.docx]

**Description of the Source Data file:**

Tabulated view of each panel of the main and supplementary figures. The individual sheets list the access information to deposited data of the metabolomics and proteomics datasets. Sheets that refer to panels displaying western-blots contain the full blots, a list with antibodies used, their working-dilutions and information on validation by the manufacturer. Sheets that refer to a panel displaying smaller datasets, such as boxplot representations of proteomic data, qPCR results, mtDNA content quantification, or line graphs such as oxygen consumption rates or survival rates directly list the data points. All other data that support findings of this study can be obtained from the corresponding authors upon reasonably request.

**Description of additional Supplementary Data files:**

Supplementary data files provide tabulated data used for metabolomics and proteomics analyses in this study. These files include metabolite changes in patient-derived fibroblasts (Supplementary Data1), protein level changes in patient-derived fibroblasts and myotubes (Supplementary Data 2 and 3), lysine succinylation sites in patient-derived fibroblasts and myotubes (Supplementary Data 4 and 5), and lysine succinylation sites in patient-derived fibroblasts overlapping with sites identified in fibroblasts from *Sirt5^-/-^* knock-out mice.

**File name: Supplementary Data 1**

**Description: Metabolite changes in patient-derived fibroblasts**

Metabolomics analyses from patient fibroblasts at day 0 and at day 5 of cell culture. Analyses were performed on fibroblast lines of the seven patients and of three control subjects. Statistical significance was determined using a student’s t-test with unequal variance. FC= fold change; D= day.

**File name: Supplementary Data 2**

**Description: Protein level changes detected from patient-derived fibroblasts**

Relative protein levels in SCL patient fibroblasts compared to control fibroblasts. (n=2 control fibroblast samples; n=3 patient-derived fibroblast samples). Statistical significance was determined user fisher exact test. Significant p- and q-values are indicated in green. p=patient; c= control.

**File Name: Supplementary Data 3**

**Description: Protein level changes detected from patient-derived myotubes**

Relative protein levels in SCL patient myotubes compared to control myotubes (n=2 control myotube samples; n=1 patient-derived myotube sample). Statistical significance was determined user fisher exact test. Significant p- and q-values are indicated in green. p=patient; c=control.

**File Name: Supplementary Data 4**

**Description: Lysine succinylation sites detected from patient-derived fibroblasts**

Succinylation sites detected in fibroblasts from SCL patients (n=2 control, n=3 SCL patient-derived fibroblasts). Statistical significance was determined using fisher exact test. The column myo_fibro_overlap indicates whether the site is succinylated in both cell lines (TRUE) or only in fibrolasts (FALSE). The column fibro_sirt5KO_overlap indicates whether the site is succinylated in fibroblasts and SIRT5 knock out conditions (TRUE) or only in fibroblasts (FALSE). FDR=false discovery rate.

**File Name: Supplementary Data 5**

**Description: Lysine succinylation sites detected from patient-derived myotubes**

Succinylation sites detected in myotubes from SCL patients (n=2 control, n=1 SCL patient-derived myoblast, 2 technical replicates). Statistical significance was determined using fisher exact test. The column myo_fibro_overlap indicates whether the site is succinylated in both cell lines (TRUE) or only in myotubes (FALSE). The column myo_sirt5KO_overlap indicates whether the site is succinylated in myotubes and SIRT5 knock out conditions (TRUE) or only in myotubes (FALSE), FDR=false discovery rate.

**File Name: Supplementary Data 6**

**Description: Lysine succinylation sites overlapping with sites described in fibroblasts from *Sirt5^-/-^* mice**

Comparison of succinylation sites from SCL patient fibroblasts and fibroblasts derived from *Sirt5* knock-out mice. Freq=frequency; indicates the number of succinylation sites on each protein.
