# Supplementary material for: SUCLA2 mutations cause global protein succinylation contributing to the pathomechanism of a hereditary mitochondrial disease
Source: Nat Commun. 2020 Nov 23;11:5927. doi: 10.1038/s41467-020-19743-4 (PMC7684291; doi:10.1038/s41467-020-19743-4)
Supplement: Supplementary file 10 — Reporting Summary [file 41467_2020_19743_MOESM10_ESM.pdf]

nature research

Corresponding author(s): Philipp Gut, Birgit Schilling, Anu Suomalainen, Eric Verdin

Last updated by author(s): 13/10/20

Reporting Summary

Nature Research wishes to improve the reproducibility of the work that we publish. This form provides structure for consistency and transparency in reporting. For further information on Nature Research policies, see Authors & Referees and the Editorial Policy Checklist .

Statistics

For all statistical analyses, confirm that the following items are present in the figure legend, table legend, main text, or Methods section.

n/a	Confirmed	
☐	☒	The exact sample size (n) for each experimental group/condition, given as a discrete number and unit of measurement
☐	☒	A statement on whether measurements were taken from distinct samples or whether the same sample was measured repeatedly
☐	☒	The statistical test(s) used AND whether they are one- or two-sided<br>*Only common tests should be described solely by name; describe more complex techniques in the Methods section.*
☐	☒	A description of all covariates tested
☐	☒	A description of any assumptions or corrections, such as tests of normality and adjustment for multiple comparisons
☐	☒	A full description of the statistical parameters including central tendency (e.g. means) or other basic estimates (e.g. regression coefficient) AND variation (e.g. standard deviation) or associated estimates of uncertainty (e.g. confidence intervals)
☐	☒	For null hypothesis testing, the test statistic (e.g. F, t, r) with confidence intervals, effect sizes, degrees of freedom and P value noted<br>*Give P values as exact values whenever suitable.*
☒	☐	For Bayesian analysis, information on the choice of priors and Markov chain Monte Carlo settings
☒	☐	For hierarchical and complex designs, identification of the appropriate level for tests and full reporting of outcomes
☒	☐	Estimates of effect sizes (e.g. Cohen's d, Pearson's r), indicating how they were calculated

Our web collection on statistics for biologists contains articles on many of the points above.

Software and code

Policy information about availability of computer code

Data collection	All proteomics MS data were collected using an Eksigent nLC system with a cHiPLC column system coupled to a SCIEX TripleTOF 6600 MS system using Sciex Analyst software version 1.7. Metabolomics data were collected on a Q Exactive Plus mass spectrometer (Thermo Scientific). Detailed information on data collection for polar and unpolar metabolites are described in the methods section.<br>Metabolomics data from zebrafish samples were collected on a Vanquish UHPLC (Thermo Fisher) coupled to an Orbitrap Fusion Lumos mass spectrometer (Thermo Fisher).
Data analysis	Codes: R-scripts used in this study can be downloaded used the following link: [https://github.com/jessegmeyerlab/SUCLA2-deficiency].<br>The following softwares were used for data analysis:<br>Prism Graphpad Version 8.4.1 (676); Discovery Studio v4.1; Maxquant v1.6.17.0; Sieve 2.0; Skyline 20.1; Spectronaut pulsar Version 11.0.15038.12.33511; R version 3.2.3; PIQED version 1.01

For manuscripts utilizing custom algorithms or software that are central to the research but not yet described in published literature, software must be made available to editors/reviewers. We strongly encourage code deposition in a community repository (e.g. GitHub). See the Nature Research guidelines for submitting code & software for further information.

Data

Policy information about availability of data

All manuscripts must include a data availability statement. This statement should provide the following information, where applicable:

- Accession codes, unique identifiers, or web links for publicly available datasets
- A list of figures that have associated raw data
- A description of any restrictions on data availability

Source data: A file with source data is available as part of the supplementary information. This file is a tabulated document with sheets corresponding to each panel of the main and supplementary figures. For large datasets, such as metabolomics and proteomics the accession codes are listed in the source data file. For small datasets, the source data is directly provided through this file. Western blot images are shown as uncropped blots accompanied by information on the antibodies

used, including their working dilution. All other raw data that support the findings of this study are available from the corresponding authors upon reasonable request.

Proteomics data from this study: Raw mass spectrometry data of protein hyper-succinylation in fibroblasts and myotubes derived from patients with mutations in succinyl-coA ligase along with supplemental tables of identifications and quantification are available from the UCSD proteomics resource Massive: [https://massive.ucsd.edu/ProteoSAFe/dataset.jsp?task=7ca3028f854e4996b58f1fa1fc2286fd]
The Skyline document containing the PTM spectral library and quantitative data is available from panorama: [https://panoramaweb.org/project/Schilling/SuccinylCoALigase/begin.view?]

Proteomics data used for comparative analyses:
Proteomics data from the Rardin et al manuscript used to determine changes in lysine succinylation of TCA cycle subunits in mouse liver can be found using the following links:
Protein quantification:
[https://www.cell.com/cms/10.1016/j.cmet.2013.11.013/attachment/74906873-3844-47b3-8cc5-a66cb052ecbf/mmc3.xlsx]
Peptide quantification:
[https://www.cell.com/cms/10.1016/j.cmet.2013.11.013/attachment/6c379a66-daf2-413f-884a-c23a478fcbae/mmc4.xlsx]
The proteomics analysis from the Park et al manuscript on changes in lysine succinylation in fibroblasts from control and Sirt5-/- mice can be found using the following link:
[https://www.cell.com/cms/10.1016/j.molcel.2013.06.001/attachment/59f72482-fad7-48fd-a9fa-9f38fd7a9232/mmc2.xls]

Metabolomics: Metabolomics data for d0 and 5 fibroblasts were deposited at Metabolomics Workbench. Data can be accessed using the following link: [http://dx.doi.org/10.21228/M8M116]

Homology modeling: The homology modelling of the human SCL heterodimer is based on the structure E.Coli Succinyl-CoA synthetase with the PDB code 1CQL. The code is accessible using this link: [https://pdbj.org/emnavi/quick.php?id=pdb-1cqi]

A data availability section in the main manuscript further details how to retrieve primary data supporting the findings of this study.

Field-specific reporting

Please select the one below that is the best fit for your research. If you are not sure, read the appropriate sections before making your selection.

☒ Life sciences ☐ Behavioural & social sciences ☐ Ecological, evolutionary & environmental sciences

For a reference copy of the document with all sections, see nature.com/documents/nr-reporting-summary-flat.pdf

Life sciences study design

All studies must disclose on these points even when the disclosure is negative.

Sample size

Sample sizes for cell culture experiments were based on the availability of cell lines from patient and control subjects. For metabolomics all seven patient-derived fibroblast lines and all three control fibroblasts were used. For metabolomics experiments on myoblasts and myotubes, the one patient-derived cell line was used and the available two control myotube lines. The myotube samples from the patient were run as technical replicate. SDS page and western blot experiments were performed on all available lines except for the fibroblast line of patient 6 because of the limited number of lanes per gel. Patient 6 shares the same mutation and clinical characteristics as patients 3 to 5.
For proteomics experiments three fibroblasts lines were chosen to represent three different types of genetic mutations in the SUCLA2 gene and all available myoblast lines were used. The number of sample size is limited by the complexity of running proteomics workflows and data analysis.
No power calculations were performed prior to zebrafish experiments. Instead, a large number of larvae was obtained from 6 to 10 crosses of parents in the respective genetic backgrounds. The larvae were collected and used as pools of 10-15 larvae to obtain sufficient amounts of tissue for SDS page and western blot experiments. Similarly, a large number of larvae were obtained for survival experiments. After collection of the clutches, experiments were performed blinded through counting of dead zebrafish, and collection followed by post-hoc genotyping. The experiments using fed conditions used numbers bewtween 77 and 82 animals, the experiments in volume restriction 21 to 24 animals.

Data exclusions

Samples from all patient-derived cell lines available were included. Six out of the seven fibroblast cell lines were analyzed with SDS-PAGE and Western blot. Fibroblasts from patient 6 were not loaded to accommodate the number of lanes per gel. Patient 6 was chosen to be left out due to the fact that patients 3 to 5 carry the same genetic mutations (c534+1g>A) and have similar clinical manifestations. No data points were excluded from data obtained as part of the zebrafish experiments.

Replication

Detection of hyper-succinylation was assessed by SDS-PAGE and western blot in fibroblast cell lines from six out of the seven patients (see section above). The western blots were repeated approximately ten times with similar results. Mass spectrometry experiment was only performed once due to the complexity of running mass spectrometry workflows and analyzing these large datasets. The large overlap between detected lysine succinylation sites detected from fibroblasts and myotubes substantiates the validity of the findings.
Survival experiments shown in Figure 5 are pooled analyses from two independent experiments. Zebrafish survival experiments in similar conditions were performed approximately four times with results confirming the findings of the manuscript. Western blots on zebrafish samples were performed once in each condition due to the difficulty obtaining sufficient numbers of zebrafish larvae for biochemical analyses. The results obtained from gain- and loss-of-function studies in zebrafish larvae and on muscle tissue from adult heterozygous animals show

	consistent hyper-succinylation in Sucla2 and Sirt5 loss of function as shown in Figure 4 and Supplementary Figure 6 and reproduce the findings in patient-derived cell lines. The consistency across different conditions and experimental models substantiates the findings and argues against sacrificing additional animals for replication of these experiments following 3R principles.
Randomization	All available patient-derived samples were analyzed with SDS-PAGE and Western blot, and were not randomized to detect possible mutation-specific differences. Samples were treated in random order for mass spec sample preparation and analysis.
Blinding	The technician preparing the mass spec samples was blind to their identities. The scientist analyzing the zebrafish survival time courses was blinded to the genetic background of the zebrafish and data were "unblinded" only through genotyping after completion of the experiment.

Reporting for specific materials, systems and methods

We require information from authors about some types of materials, experimental systems and methods used in many studies. Here, indicate whether each material, system or method listed is relevant to your study. If you are not sure if a list item applies to your research, read the appropriate section before selecting a response.

Materials & experimental systems

n/a	Involved in the study	
☐	☒	Antibodies
☐	☒	Eukaryotic cell lines
☒	☐	Palaeontology
☐	☒	Animals and other organisms
☐	☒	Human research participants
☒	☐	Clinical data

Methods

n/a	Involved in the study	
☒	☐	ChIP-seq
☒	☐	Flow cytometry
☒	☐	MRI-based neuroimaging

Antibodies

Antibodies used	A list with antibodies and working solutions can be found in the supplementary information, Table 1 as well as in the source data file. Pan anti-succinyllysine antibody (working dilution 1/1000), SUCLA2 (working dilution 1/1000), TUBULIN (working dilution 1/10000), HSC-70 (working dilution 1/5000), GAPDH (working dilution 1/1000), PTMScan succinyl-lysine motif [Succ-K] Kit (working dilution 1/1000).
Validation	All antibodies have been widely used in the research community. A full list with all references to catalogue numbers is provided in Table 1 of the supplementary information. The source data file lists information from the manufacturers websites, including species reactivities, recommended dilutions, validation blots, and references.

Eukaryotic cell lines

Policy information about cell lines

Cell line source(s)	Fibroblast and myoblast lines were obtained from soft tissue and muscle biopsies, taken for diagnostic purposes from patients with mitochondrial disease due to disease-causing mutations in SUCLA2. The cell lines were authenticated by sequencing analysis to confirm the mutations, followed by biochemical analyses to characterize bioenergetic deficits. These lines were previously published with the exception of line from patient 7, as shown in table 1 of the manuscript.
Authentication	Cell lines were established and characterized in author's laboratory and the majority of these lines have been published previously (see table 1 and references section of the manuscript).
Mycoplasma contamination	All patient-derived cell lines were tested negative for mycoplasma.
Commonly misidentified lines<br>(See ICLAC register)	No commonly misidentified lines were used in this study.

Animals and other organisms

Policy information about studies involving animals; ARRIVE guidelines recommended for reporting animal research

Laboratory animals	Wild-type AB zebrafish lines were used as controls as well as for the generation of genetically modified lines. The following lines were generated for the purpose of this study: sucla2-/- (nei010) mutant zebrafish ,<br>sirt5-/- (nei004) mutant zebrafish, and Tg(ubi:sirt5;cryaaZsGreen1) (nei005) transgene carrying zebrafish. All lines have been characterized extensively and will be made available to the community through stock centers.<br>Experiments in zebrafish larvae were performed at 7dpf. The timecourse was performed up to 14 dpf. Indication of the gender is not applicable at these stages as sexual maturation occurs at post larval stages. Adult zebrafish experiments where performed at mid-adult stages at 4 months of age using zebrafish from both sexes at comparable numbers. No considerable difference in succinylation has been observed between genders.
Wild animals	This study did not involve the use of wild animals.

Field-collected samples	This study did not involve field-collected samples.
Ethics oversight	All experimental procedures were carried out according to the Swiss and EU ethical guidelines and were approved by the animal experimentation ethical committee of Canton of Vaud (permits VD-H13 and VD3177).

Note that full information on the approval of the study protocol must also be provided in the manuscript.

Human research participants

Policy information about studies involving human research participants

Population characteristics	Human research participants are pediatric patients admitted to clinics for diagnostics of mitochondrial disease. Each patient has been identified to have disease-causing genetic defects in SUCLA2 (homozygous Asp333Gly, compund heterozygous Asp333Gly/13q14 deletion, homozygous c. 534+1G>A or homozygous Arg407Leu). The patient covariants are described in Table 1 of the manuscript.
Recruitment	Patients were admitted to clinics for examination and treatment for mitochondrial disease. SCL deficiency is a rare disease. All patients with deficiencies in SUCLA2 that consented were included into the study.
Ethics oversight	Ethics committee for Paediatrics, Adolescents and Psychiatry of Helsinki University Hospital.

Note that full information on the approval of the study protocol must also be provided in the manuscript.
